# *Roseomonas mucosa*-Induced Peritonitis in a Patient Undergoing Continuous Cycler Peritoneal Dialysis: Case Report and Literature Analysis

**DOI:** 10.1155/2021/1979332

**Published:** 2021-11-01

**Authors:** Sasmit Roy, Sumit Patel, Hardhik Kummamuru, Amarinder Singh Garcha, Rohan Gupta, Sreedhar Adapa

**Affiliations:** ^1^Department of Medicine, Centra Health, Lynchburg, VA 24501, USA; ^2^Department of Internal Medicine, Adventist Health Community Care, Hanford, CA 93230, USA; ^3^University of Texas, Arlington, TX 76019, USA; ^4^Kaweah Delta Health Care District, Visalia, CA 93291, USA; ^5^Community Regional Medical Center, Fresno, CA 93721, USA

## Abstract

*Roseomonas* species, a rare Gram-negative microorganism, has seldom been reported to cause peritonitis in end-stage renal disease patients on peritoneal dialysis. Only seven cases of peritonitis by this rare microorganism have been reported worldwide. Treatment options can be challenging if not detected early and can lead to significant morbidity and mortality along with the switching of the dialysis modality to hemodialysis which is highly undesirable. Our patient is a 65-year-old Caucasian female who needed to be changed to emergency hemodialysis due to inability to perform peritoneal dialysis from suspected peritonitis and was subsequently discovered to have peritonitis from *Roseomonas mucosa*. She recovered with a prolonged antibiotics course and returned to peritoneal dialysis in 3 months following her treatment completion. Prompt diagnosis and prolonged antibiotics are a cornerstone in the management of this rare microorganism to prevent mortality and morbidity from peritonitis.

## 1. Introduction

Peritonitis, caused by infectious microorganisms, is a hazardous outcome in end-stage renal disease (ESRD) patients undergoing peritoneal dialysis. It can lead to a high rate of morbidity and mortality if not treated properly and timely [1]. *Roseomonas* species, a rare Gram-negative, pink pigmented, oxidized coccobacillus, is normally identified as environmental commensal in water and soil and was first reported as infectious etiology in clinical medicine by Rihs et al. in 1993 [2]. First reported case of *Roseomonas* peritonitis was documented by JA Sandoe et al. in 1997 [3]. Only a handful of cases of *Roseomonas*-induced peritonitis in peritoneal dialysis patients have been reported worldwide, seven to be exact [3–9]. The most affected mode of infection can be bloodstream, musculoskeletal, skin, and soft tissue with peritonitis being extremely rare [10]. The treatment can be challenging if proper antibiotics are not initiated timely and thus may delay the course of clinical recovery and can even lead to serious ill-fated consequences.

## 2. Case Report

A 65-year-old Caucasian female patient who was on peritoneal dialysis (PD) for end-stage renal disease (ESRD) presented to the emergency room (ER) with shortness of breath for 3 days. The PD catheter stopped draining for the last 5 days, and the patient was waiting to see the surgeon adjust the position of it. The medical history was significant for hypertension, hyperlipidemia, and ESRD. She had no recent history of going anywhere for swimming or taking bath in a hot tub. Home medication included amlodipine 10 mg daily, clonidine 0.1 mg three times a day, furosemide 40 mg daily, metoprolol succinate 50 mg daily, renal vitamin 1 tablet daily, pravastatin 10 mg at nighttime, and potassium chloride 20 milliequivalents daily. She was doing continuous cycler peritoneal dialysis at home with a total of 4 exchanges of 2.5% dextrose solution bags, each exchange for 2 hours and with 2 Liters fill during the exchanges. Her total PD duration was 9 hours at night with no daytime dwell. She was doing PD for the last 2 years with no issues of infection before. Her PD was not accompanied by any major issues of constipation or diarrhea in recent past. There was no major social shift in home dynamics, with her primary care giver, i.e., her husband staying with her since she started PD and helping her with the treatment throughout. She did not have any pets at home neither did she have any recent visit from any sick relatives or any pets from neighbors or friends.

Vital signs revealed a temperature of 97.7 Fahrenheit (F), pulse rate of 82 beats per minute (bpm), blood pressure of 170/106 mm Hg, and respiratory rate of 24 breaths per minute with oxygen saturation of 90% on room air. Physical examination showed that the patient was in visible respiratory distress. Lung examination was significant for decreased air entry in bilateral lung fields and basal crackles, and the patient had bilateral pitting pedal edema. The rest of the physical examination was insignificant. Initial admission labs are as given in Table 1.

Attempts to access the PD catheter in ER were unsuccessful with no drainage of dialysate. Because of hyperkalemia and significant volume overload, she underwent emergent hemodialysis after getting a temporary left internal jugular dialysis catheter placed urgently. General surgery was consulted for the malfunctioning PD catheter, and the patient underwent diagnostic laparoscopy. The PD catheter was noted to be clogged with fibrin glue and mispositioned to the right lower quadrant. The catheter was unclogged followed by repositioning to the most dependent part of the peritoneal cavity in the pelvis. The peritoneal fluid was noted to be cloudy, and the fluid was sent for cell count, Gram stain, and culture. The patient was also started on empiric intraperitoneal (IP) vancomycin and aztreonam for suspected peritonitis. PD fluid cell count revealed white blood cell (WBC) count of 209/microliter (*µ*L) with 72% neutrophils and red blood cells (RBC) count of 2337/*µ*L, and Gram stain revealed >100 WBC/low power field (lpf) with no organism. Aerobic bottle grew Gram-negative bacilli at 48 hours, and the organism was identified as *Roseomonas mucosa* at 96 hours by matrix-assisted laser desorption/ionization-time of flight (MALDI-TOF) mass spectrometry (MS). The organism was resistant to cephalosporin and sensitive to gentamicin. After clinical stability in 3 days, the patient was discharged from the hospital with intraperitoneal gentamicin for a total of 3 weeks. The patients continued to have drainage issues and persistently elevated PD fluid WBC counts despite being on antibiotics. The PD catheter was removed, and the modality was changed to hemodialysis. 3 months after the episode, she reverted to peritoneal dialysis again. She continues to be on PD now with no recurrence of peritonitis from *Roseomonas* or any other species as per subsequent serial negative PD fluid cultures.

## 3. Discussion

Peritonitis, described as an inflammation of the peritoneum, is a serious complication for ESRD patients undergoing PD and is usually caused by bacteria or fungi. Peritonitis maybe secondary to nondialytic-related systemic or intraabdominal processes or related to peritoneal dialysis itself. Tzamaloukas AH et al. in one review article demonstrated that less than 6% of cases are related to secondary causes, while the rest 94% cases are due to peritoneal dialysis itself in patients undergoing continuous ambulatory peritoneal dialysis (CAPD) [11]. Common clinical features are abdominal pain with cloudy effluent, fever, nausea/vomiting, and hypotension. Common diagnostic methods employed are to obtain cell type and count and peritoneal fluid culture. The major laboratory parameter that is highly suggestive of bacterial infection is peritoneal fluid leukocyte count above 100 cells/mm^3^. Less than 8 leucocytes/mm^3^ go against a diagnosis of peritonitis [12]. Peritoneal fluid culture is positive in 80–95% of cases of peritonitis if proper technique is performed [13].

Most of the peritonitis is caused by bacteria. 45–65% of bacteria being a Gram-positive organism, while 15–35% representing Gram-negative ones [14, 15]. Coagulase-negative *Staphylococcus* is the commonest cause of peritonitis. In one of the most recent extensive studies by Whitty et al., *Staphylococcus* accounted for 60% of infectious etiology by Gram-positive organisms and 39% overall causative organisms [16]. *Streptococcus* species caused 20% of infection, while other common Gram-positive organisms were *Staphylococcus aureus*, *Enterococcus*, and *Corynebacterium* spp. Common Gram-negative organisms are *Escherichia coli*, *Pseudomonas*, and *Klebsiella* species [16]. Among other species, fungal infection with* Candida albicans* and *Candida parapsilosis* is becoming more and more prevalent [17].

As per the 2016 International Society of Peritoneal Dialysis (ISPD) guidelines [18], initial empiric treatment includes a combination of vancomycin or first generational cephalosporins such as cefazolin and third or fourth generational cephalosporins such as ceftazidime or cefepime or an aminoglycoside such as gentamicin or aztreonam to cover both Gram type species. Though commonly not needing removal of the dialysis catheter if infected by Gram-negative organisms, sometimes, rare infections occur resulting in a change in modality of dialysis to hemodialysis or the removal of the catheter [19]. Worse complications of PD could result in hospitalization, morbidity, or death [19].

In a study concluded recently, the incidence rate for peritonitis episodes was found through observation in a large-scale patient population worldwide [20]. This was conducted in 209 facilities across seven countries (New Zealand, Australia, Canada, Japan, Thailand, the United States, and the United Kingdom). Here, it was reported that out of 7,051 patients on peritoneal dialysis, a count of 2,272 episodes of peritonitis was recorded in total. This calculates to be a rate of about 0.28 per patient per year. In the United States, it is reported as 0.26 episodes per patient per year, with a given range of 0.24–0.27 episodes.

*Roseomonas* is a pink segmented, coccoid rod. It is a Gram-negative bacterium that has four named subspecies, known as *R. gilardii, R. cervicalis, R. mucosa*, and *R. fauriae* [3]. This was first reported by Rihs et al. [2] in 1993 as a species different from the initial thought genus of *Methylobacterium*. *Roseomonas* is found naturally and is accessible through water and soil. Reported infection through *Roseomonas* has been documented in bloodstream, as well as in cerebrospinal fluid, skin and soft tissues [10]. The route of *Roseomonas* infection is uncertain. It is theorized that the infection could occur through water, such as water from contaminated faucets. There are very few cases of peritonitis in PD patients that have been reported with *Roseomonas* as a causative organism. As per PubMed Central literature search, worldwide there have been only seven recorded cases with the different subspecies of *Roseomonas* infection in PD patients [3–9]. They are summarized in Table 2. The first reported case in PD patients came out of the UK by Sandoe et al. in 1997. Out of the seven patients recorded for *Roseomonas* in PD, three patients were affected by *R. gilardii*, one with *R. fauriae*, one with *Roseomonas* genus, and two with *R. mucosa*. Out of the 7 cases, 4 cases were on CAPD, while 3 were on CCPD.

Treatment methods for *Roseomonas* peritonitis are broad-spectrum antibiotics, given through intraperitoneal administration. In a systemic review of all published *Roseomonas* cases recently conducted by Ioannou et al. [10], beta-lactams resistance was discovered to be significantly elevated with respective penicillin, piperacillin/tazobactam, and cephalosporin resistance being at 96.6%, 90.7%, and 77.8%. Carbapenems and quinolones are most effective in treating this rare organism [10]. Overall mortality to *Roseomonas* species was reported by them to be at 1%. Because it is still a rare organism and due to paucity of clinical evidence, it is suggested that treatment duration be stretched to 3 weeks of antibiotics [8]. Hopefully, this will prevent the change in modality to hemodialysis which is always undesirable to patients.

## 4. Conclusions

Due to the rarity of this microorganism causing infectious peritonitis and the high rate of resistance to cephalosporin which is considered a first-time empiric therapy in peritonitis management, *Roseomonas* species needs to be inculcated in medical literature more. This will help nephrologists broaden their differential of peritonitis, especially in cases of nonresolving clinical peritonitis.

## Figures and Tables

**Table 1:** Admission labs.

Lab values	Reference range	On presentation
Sodium (millimole/liter)	135–145	131
Potassium (millimole/liter)	3.5–5.1	7.1
Chloride (millimole/liter)	98–106	100
CO_2_ (millimole/liter)	23–29	22
Blood urea nitrogen (BUN) (milligram/deciliters)	8–24	119
Creatinine (milligrams/deciliters)	0.7–1.3	9.43
Calcium (milligram/deciliters)	8.8–10.2	9.3
Glucose (milligram/deciliters)	70–105	99
White blood cells (thousand/millimiters^3^)	4–10	6.9
Hemoglobin (gram/deciliters)	14–16	10.6
Platelets (thousand/millimiters^3^)	150–450	183

**Table 2:** Summary of all cases reported to have *Roseomonas* spp. peritonitis with outcome.

Author	Year	Patient age/gender	Study country	*Roseomonas* species	Dialysis modality	Dialysis culture	Treatment received	Outcome	Modality changed
Sandoe et al. [3]	1997	62/F	UK	*R. gilardii*	CAPD	Positive	IP vancomycin and then IP netilmicin	Resolved	No
Bibashi et al. [4]	2000	65/F	Greece	*R. fauriae*	CAPD	Positive	IP vancomycin plus IP netilmicin and then ciprofloxacin	Resolved	No
Tsai et al. [5]	2012	48/M	Taiwan		CCPD	Positive	IP ceftazidime plus IP cefazolin and then PO ciprofloxacin	Resolved	No
Boyd et al. [6]	2012	19/M	USA	*R. mucosa*	CCPD	Positive	IP ceftazidime and then IP ciprofloxacin	Resolved	No
Matsukuma et al. [7]	2014	61/M	Japan	*R. mucosa*	CAPD	Positive	IP ceftazidime and then IP ciprofloxacin	Resolved	Yes, to HD
Malini et al. [8]	2016	61	Malaysia	*R. gilardii*	CAPD	Positive	IP ceftazidime plus IP cloxacillin and then IP meropenem	Resolved	Yes, to HD
Burstahler et al. [9]	2021	73/M	Russia	*R. gilardii*	CCPD	Positive	IP ceftazidime plus IP vancomycin and then IP ciprofloxacin	Resolved	No

F, female; M, male; R, *Roseomonas*; UK, the United Kingdom; USA, the United States of America; IV, intravenous; IP, intraperitoneal; PO, per oral; CAPD, continuous ambulatory peritoneal dialysis; CCPD, continuous cycler peritoneal dialysis; HD, hemodialysis.

## Data Availability

The data used to support the findings of this study are collected from PubMed and Google Scholar databases and are included within the article.

## References

[1] Adapa S., Naramala S., Boken D., Moreno A., Konala V. M. (2019). Peritonitis from anaerobic gram-positive cocci likely due to translocation of bacteria from gut in a patient undergoing peritoneal dialysis. *Cureus*.

[2] Rihs J. D., Brenner D. J., Weaver R. E., Steigerwalt A. G., Hollis D. G., Yu V. L. (1993). Roseomonas, a new genus associated with bacteremia and other human infections. *Journal of Clinical Microbiology*.

[3] Sandoe J. A., Malnick H., Loudon K. W. (1997). A case of peritonitis caused by Roseomonas gilardii in a patient undergoing continuous ambulatory peritoneal dialysis. *Journal of Clinical Microbiology*.

[4] Bibashi E., Sofianou D., Kontopoulou K., Mitsopoulos E., Kokolina E. (2000). Peritonitis due to *Roseomonas fauriae* in a patient undergoing continuous ambulatory peritoneal dialysis. *Journal of Clinical Microbiology*.

[5] Tsai S.-F., Chen C.-H., Shu K.-H., Wu M.-J. (2012). Peritonitis caused by Roseomonas in a patient undergoing automated peritoneal dialysis: case report and literature review. *Internal Medicine*.

[6] Boyd M. A., Laurens M. B., Fiorella P. D., Mendley S. R. (2012). Peritonitis and technique failure caused by Roseomonas mucosa in an adolescent infected with HIV on continuous cycling peritoneal dialysis. *Journal of Clinical Microbiology*.

[7] Matsukuma Y., Sugawara K., Shimano S. (2014). A case of bacterial peritonitis caused by Roseomonas mucosa in a patient undergoing continuous ambulatory peritoneal dialysis. *CEN Case Reports*.

[8] Malini S., Goh B. L., Lim T. S. (2016). A rare case of Roseomonas gilardii peritonitis in a patient on continuous ambulatory peritoneal dialysis. *Peritoneal Dialysis International: Journal of the International Society for Peritoneal Dialysis*.

[9] Burgstahler M. S., Bieber S. D., Pfeiffer D. C. (2021). A case of *Roseomonas gilardii* peritonitis associated with a flooded peritoneal dialysis treatment space. *Annals of Clinical and Laboratory Science*.

[10] Ioannou P., Mavrikaki V., Kofteridis D. P. (2020). *Roseomonas* species infections in humans: a systematic review. *Journal of Chemotherapy*.

[11] Tzamaloukas A. H., Obermiller L. E., Gibel L. J. (1993). Peritonitis associated with intra-abdominal pathology in continuous ambulatory peritoneal dialysis patients. *Peritoneal Dialysis International*.

[12] Tranaeus A., Heimbürger O., Lindholm B. (1989). Peritonitis in continuous ambulatory peritoneal dialysis (CAPD): diagnostic findings, therapeutic outcome and complications. *Peritoneal Dialysis International: Journal of the International Society for Peritoneal Dialysis*.

[13] Li P. K.-T., Szeto C. C., Piraino B. (2010). Peritoneal dialysis-related infections recommendations: 2010 update. *Peritoneal Dialysis International: Journal of the International Society for Peritoneal Dialysis*.

[14] Mujais S. (2006). Microbiology and outcomes of peritonitis in North America. *Kidney International*.

[15] Oo T. N., Roberts T. L., Collins A. J. (2005). A comparison of peritonitis rates from the United States Renal Data System database: CAPD versus continuous cycling peritoneal dialysis patients. *American Journal of Kidney Diseases*.

[16] Whitty R., Bargman J. M., Kiss A., Dresser L., Lui P. (2017). Residual kidney function and peritoneal dialysis-associated peritonitis treatment outcomes. *Clinical Journal of the American Society of Nephrology*.

[17] Roy S., Vantipalli P., Garcha A., Pokal M., Adapa S. (2021). The emerging uncommon non-albicans Candida: Candida parapsilosis peritonitis in a peritoneal dialysis patient. *Cureus*.

[18] Li P. K.-T., Szeto C. C., Piraino B. (2016). ISPD peritonitis recommendations: 2016 update on prevention and treatment. *Peritoneal Dialysis International: Journal of the International Society for Peritoneal Dialysis*.

[19] Adapa S., Naramala S., Madhira B. R., Gayam V., Sahasranam P., Konala V. M. (2019). Peritonitis secondary to uncommon gram-negative coccobacillus transmitted from a cat in a patient on peritoneal dialysis. *Journal of Investigative Medicine High Impact Case Reports*.

[20] Perl J., Fuller D. S., Bieber B. A. (2020). Peritoneal dialysis-related infection rates and outcomes: results from the peritoneal dialysis outcomes and practice patterns study (PDOPPS). *American Journal of Kidney Diseases*.

